# Insulin-like growth factor binding protein-3 inhibits monocyte adhesion to retinal endothelial cells in high glucose conditions

**Published:** 2013-04-05

**Authors:** Qiuhua Zhang, Youde Jiang, Jordan J. Toutounchian, Carl Soderland, C. Ryan Yates, Jena J. Steinle

**Affiliations:** 1 Department of Ophthalmology, University of Tennessee Health Science Center, Memphis, TN; 2 Department of Anatomy & Neurobiology, University of Tennessee Health Science Center, Memphis, TN; 3 Department of Pharmaceutical Sciences, University of Tennessee Health Science Center, Memphis, TN; 4 Department of Cell Systems Corp, Kirkland, WA

## Abstract

**Purpose:**

Insulin-like growth factor binding protein-3 (IGFBP-3) is cytoprotective in the retina. The goal of this study was to investigate whether IGFBP-3 inhibits monocyte-endothelial cell adhesion associated with hyperglycemia.

**Methods:**

Human retinal vascular endothelial cells (RECs) were grown in normal (5 mM), medium (15 mM), or high glucose medium (25 mM) for 72 h. After 48 h, cells were transfected with endothelial-cell-specific, non-IGF binding IGFBP-3 plasmid DNA (IGFBP-3NB) at 1 μg/ml for 24 h. Cells were serum starved for 16 h and treated with tumor necrosis factor-alpha (TNF-α; 10 ng/ml) for 4 h. Cell proteins were extracted and analyzed for intercellular adhesion molecule-1 (ICAM-1) expression with enzyme-linked immunosorbent assay. Additional RECs were plated onto attachment factor-coated slides, grown to 90% confluence in high glucose medium, and transfected with IGFBP-3 NB plasmid DNA or ICAM-1 small interfering RNA before treatment with or without TNF-α (10 ng/ml) for 4 h. Slides were then mounted in a parallel-plate flow chamber and subjected to a continuous flow of U937 human monocytes (10^5^/ml) in culture medium at shear stresses of 2 dynes/cm^2^, with continual exposure to TNF-α.

**Results:**

In high ambient glucose, overexpression of IGFBP-3 in RECs significantly decreased ICAM-1 expression when compared to the TNF-α-treated samples, whereas TNF-α increased monocyte-endothelial cell adhesion. IGFBP-3 significantly decreased monocyte adhesion to RECs in the high glucose condition. RECs transfected with ICAM-1 siRNA also had a decreased number of monocytes attached compared with the scrambled siRNA control.

**Conclusions:**

Data suggest that IGFBP-3 reduces monocyte-endothelial cell adhesion through decreased ICAM-1 levels in a hyperglycemic environment. This is the first demonstration of the role of IGFBP-3 in inhibiting monocyte-endothelial cell adhesion.

## Introduction

Diabetic retinopathy remains the most common cause of vision impairment in working-age adults in the United States [1]. The progression of diabetic retinopathy is a complex multistep process that starts from endothelial dysfunction and increased vascular permeability, which leads to severe vascular closure and growth of new blood vessels in the retina [2]. Inflammation plays an important role at all stages of diabetes [3-6], particularly diabetic retinopathy [7]. Tumor necrosis factor-alpha (TNF-α) is overexpressed in the adipose tissues of patients with diabetes and was identified as the first molecular link between inflammation and diabetes [8]. Additionally, work in TNF receptor I (TNF-RI)-deficient mice and TNF-RII-deficient mice showed that inhibition of TNF-α can prevent the retinal complications of diabetes [9]. Our studies have shown TNF-α is key in pathways that regulate insulin receptors and inflammatory mediators [10-12]. Understanding the activity of TNF-α on the retinal endothelium may offer a novel way to slow retinal damage induced by diabetes.

Researchers have shown that activating TNF-α signaling can inhibit insulin-like growth factor-1 (IGF-1) signaling, since silencing of IGF-1 may elevate inflammatory cytokines [13,14]. The IGF family plays a key role in the process of diabetic retinopathy [15]. Abundant in vivo animal evidence and data from in vitro cell models suggest that the IGF family is a multicomponent network of molecules involved in regulating pathophysiological growth processes in diabetic retinopathy [11,16,17]. IGF-binding proteins (IGFBPs) stabilize the IGFs through the formation of IGF/IGFBP complexes. IGFBP-3 is the most abundant IGFBP in the blood and was described as having “IGF-independent” functions [18]. Studies have suggested that IGFBP-3 can protect the vasculature in the retina [19-21]. We have shown that IGFBP-3 has the ability to inhibit apoptosis in high ambient glucose in vitro and in vivo [22].

In animal and human diabetic retinopathy models, researchers found that monocytes adhere and transmigrate through the endothelium [23,24]. Adhesion molecules, especially intercellular adhesion molecule 1 (ICAM-1, CD54) and vascular cell adhesion molecule 1 (VCAM-1), are involved in monocyte attachment and transmigration [25,26]. ICAM-1, a type I transmembrane glycoprotein, is associated with adhesion and transmigration of monocytes in the retina and in other vascular systems [27]. In diabetic retinopathy, high levels of TNF-α may trigger expression of retinal vascular ICAM-1 and monocyte infiltration and contribute to many pathological lesions [28]. Reports have shown that retinal vascular endothelial cells (RECs) and monocytes can secrete IGFBP-3 in addition to the circulating IGFBP-3 originating from the liver [21,29]. Recent studies have provided evidence for a potential interaction between IGFBPs and the inflammatory signaling cascades [30,31]. Here we used the IGFBP-3 plasmid that does not bind IGF-1 for transfection to detect IGFBP-3-only actions in retinal inflammation. We questioned whether IGFBP-3 plays a role in the adhesion between RECs and monocytes in the retina. We describe novel actions of IGFBP-3, suggesting that IGFBP-3 influences adhesion in the REC model as well as in the flow chamber. We also confirmed an inhibitory effect of IGFBP-3 on monocyte adhesion to the REC monolayer, providing a new molecular mechanism of IGFBP-3 actions in cellular adhesion in diabetic retinopathy.

## Methods

### Reagents

IGFBP-3 antibodies were purchased from Upstate (Lake Placid, NY). Actin antibodies were purchased from Santa Cruz Biotechnology (Santa Cruz, CA). An ICAM-1 enzyme-linked immunosorbent assay (ELISA) kit was purchased from Millipore (Bilerica, MA). The TNF-α ELISA kit was purchased from Thermo Scientific (Rockford, IL). Human ICAM-1 small interfering RNA (siRNA) and non-targeting siRNA number 1 were purchased from Dharmacon RNAi Technologies (Chicago, IL). Lipofectamine RNAiMAX Transfection Reagent and Lipofectamine 2000 were purchased from Invitrogen (Carlsbad, CA). Secondary antimouse and antirabbit antibodies conjugated with horseradish peroxidase were purchased from Promega (Madison, WI). Enhanced chemiluminescence for immunoblot development and signal detection was purchased from Amersham Biosciences (Piscataway, NJ). Recombinant TNF-\ was purchased from R&D Systems (Minneapolis, MN). IGFBP-3 NB (endothelial-cell specific IGFBP-3 plasmid that does not bind IGF-1) plasmid DNA was a gift from Dr. Maria Grant (University of Florida).

### Cell culture

Primary human RECs were acquired from Cell System Corporation (CSC, Kirkland, WA). Cells were grown in M131 medium containing microvascular growth supplements (MVGSs; Invitrogen), 10 μg/μl gentamycin, and 0.25 μg/μl amphotericin B. No serum is used for the RECs when MVGSs are used. Cells were transferred to high glucose (25 mM) medium (Cell Systems) or kept in 5 mM or 15 mM glucose or 25 mM mannitol, and supplemented with MVGSs and antibiotics for 3 days. Only primary cells up to passage 6 were used. Cells were quiesced by incubating in high or normal glucose medium without growth factors for 16 h and used to perform the experiments unless otherwise indicated.

U937 cells (human monocytes; American Type Culture Collection [ATCC]; Manassas, VA) were grown in Rosewell Park Memorial Institute 1640 medium containing 10% fetal bovine serum (FBS; Invitrogen) and antibiotics. Cell passing was performed by centrifugation with subsequent resuspension at 2×10^5^ cells/ml.

### Transfection

RECs were transfected with siRNA at a final concentration of 20 nM using RNAiMAX transfection reagent or plasmid DNA at 1 μg/μl using Lipofectamine 2000 according to the manufacturer’s instructions. The cells were used for experiments 24–48 h after transfection.

### Enzyme-linked immunosorbent assay analysis

TNF-α levels were measured using a TNF-α ELISA assay kit according to the manufacturer’s instructions following transfection with IGFBP-3NB plasmid DNA under normal or high glucose conditions. ICAM-1 levels were detected using the ICAM-1 ELISA kit after the IGFBP-3NB plasmid DNA transfection and/or TNF-α treatment. For all ELISA analyses, equal protein amounts were loaded into each well, allowing for comparisons using optical density.

### Western blot analysis

After appropriate treatments and rinsing with cold phosphate-buffered saline (PBS; 0.137 M NaCl, 0.0027 M KCl, and 0.0119 M Na_2_HPO_3_, 0.2 mm H_2_O_2_), RECs were collected in lysis buffer containing the protease, and phosphatase inhibitors were added to the RECs, and cell lysates were scraped into tubes. Equal amounts of protein from the cell or tissue extracts were separated on the precast Tris-Glycine Gel (Invitrogen), blotted onto a nitrocellulose membrane. After blocking in TBST (10 mM Tris-HCl buffer, pH 8.0, 150 mM NaCl, 0.1% Tween-20) and 5% (w/v) bovine serum albumin, the membrane was treated with anti-IGFBP-3 antibodies (1:500) followed by incubation with horseradish peroxidase–labeled secondary antibodies (1:5000). The antigen–antibody complexes were detected using a chemiluminescence reagent kit (Thermo Scientific). Mean densitometry of immunoreactive bands was assessed using Kodak software (Carestream Health, Rochester, NY), and results were expressed in densitometric units and compared to control groups for each individual experiment.

### Cell adhesion assays

The adhesive interactions between the U937 cells and the RECs were quantified using a parallel-plate flow chamber system (CytoDyne, La Jolla, CA). The RECs were plated onto attachment factor-coated slides, and grown to 90% confluence in M131 completed medium containing 25 mM glucose. Slides were then mounted in a parallel-plate flow chamber and subjected to a continuous flow of U937 human monocytes (10^5^/ml) in culture medium at shear stresses of 2 dynes/cm^2^ [32,33]. Phase contrast images of stationary/adherent U937 cells on RECs were obtained using a Nikon Diaphot 300 (Melville, NY) phase-contrast microscope equipped with a Dage-MTI series 68 camera (Michigan City, IN). Continuous flow was maintained for the duration of the experiment, and adherent cells were observed and counted at the second hour.

### Statistical analysis

All the experiments were repeated in triplicate, and the data are presented as mean±standard error of the mean (SEM). Data were analyzed with the Kruskal–Wallis non-parametric test followed by Dunn’s test with p values <0.05 considered statistically significant. In the case of western blotting, one representative blot is shown. A representative still image captures the extent of leukocyte adhesion from all experimental groups.

## Results

### Tumor necrosis factor-alpha induced intercellular adhesion molecule-1 protein levels in high ambient glucose

**Figure 1:**
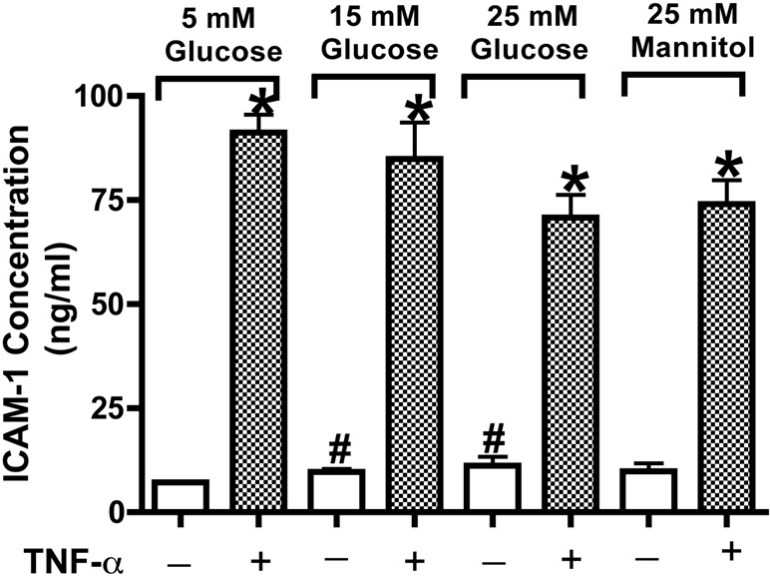
Tumor necrosis alpha (TNF-α) increased intracellular adhesion molecule 1 (ICAM-1) expression in REC in 5 mM, 15 mM, and 25 mM glucose and 25 mM mannitol concentrations. Results presented are for sICAM1 levels in RECs following treatment with TNF-α (10 ng/ml) for 4 h in normal or high ambient glucose or high osmolarity. *p<0.05 versus control. #p<0.05 versus TNF-α treatment in same glucose concentration. n=4, data are mean±SEM.

We have previously shown that TNF-α is increased in high glucose [7,34]. Since TNF-α can increase ICAM-1 levels, we wanted to evaluate whether ICAM-1 was increased in RECs grown in high glucose. Our results showed that ICAM-1 levels were increased regardless of glucose levels, while TNF-α further increased ICAM-1 levels after 4 h incubation (Figure 1). The 25 mM mannitol concentration did not influence the ICAM-1 level compared with the 5 mM glucose concentration.

### Insulin-like growth factor binding protein-3 inhibited tumor necrosis factor-alpha and intercellular adhesion molecule-1 expression in high ambient glucose

**Figure 2:**
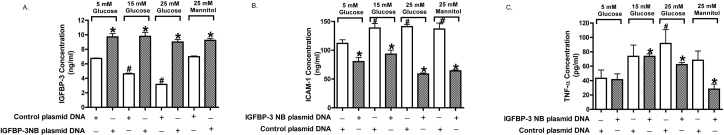
Insulin-like growth factor binding protein-3 (IGFBP-3) overexpression inhibited TNF-α and ICAM-1 expression in high glucose medium. For all panels, RECs were grown in 5 mM, 15 mM, or 25 mM glucose or 25 mM mannitol medium and transfected with either control or IGFBP-3NB plasmid DNA for 24 h. **A**: Results show IGFBP-3 levels using Western blot analyses to verify NIGFBP-3NB plasmid. **B**: Results of ICAM-1 ELISA for IGFBP-3 NB plasmid DNA transfection in REC in normal and high glucose. **C**: Results of TNF-α ELISA for IGFBP-3NB plasmid DNA transfection in REC in normal and high glucose. #p<0.05 versus 5 mM plasmid DNA; *p<0.05 versus control plasmid DNA. n=4, data are mean±SEM.

To determine a potential mechanism for IGFBP-3 inhibition of monocyte-endothelial cell adhesion, we tested the effects of IGFBP-3 on inflammatory proteins in normal and high ambient glucose. We cultured RECs in 5 mM, 15 mM, or 25 mM glucose or 25 mM mannitol medium and transfected the cells with control or IGFBP-3NB plasmid DNA for 24 h. We found that IGFBP-3 decreased TNF-α (Figure 2C) and ICAM-1 (Figure 2B) levels in high ambient glucose. The image in Figure 2A demonstrates the successful transfection of IGFBP-3NB into RECs.

### Cross talk between insulin-like growth factor binding protein-3 and tumor necrosis factor-alpha activated intercellular adhesion molecule-1 signaling cascades in retinal vascular endothelial cells in high ambient glucose

**Figure 3:**
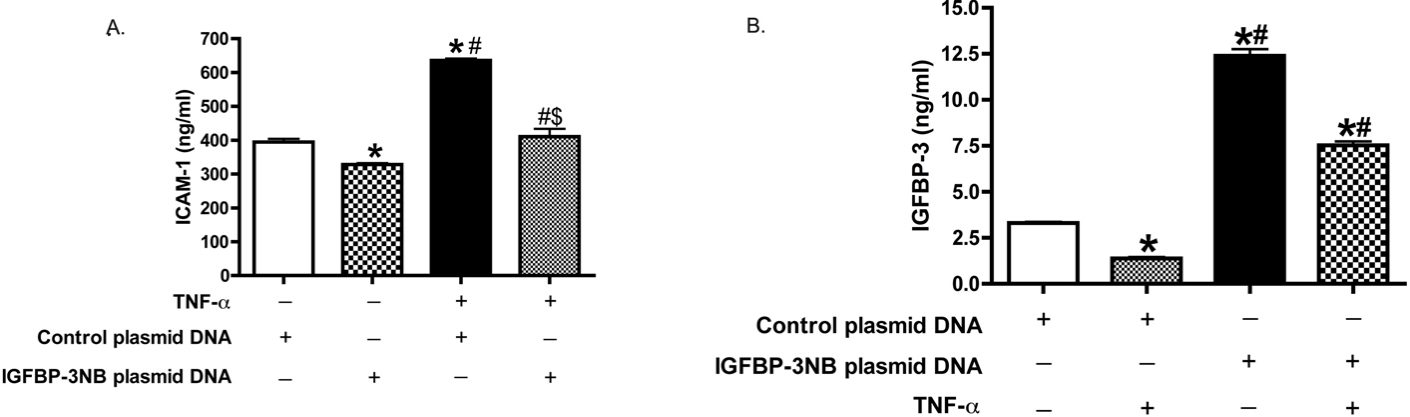
Insulin-like growth factor binding protein-3 (IGFBP-3) decreased intracellular adhesion molecule 1 (ICAM-1) expression in REC in high ambient glucose. ELISA results for IGFBP-3 (**B**) and ICAM-1 levels (**A**) in REC transfected with control or IGFBP-3 NB plasmid DNA for 24 h, following treatment with or without 10 ng/ml TNF-α for 4 h in high glucose (25 mM) medium. Equal protein was loaded for all samples, and data are presented as optical density measurements. *p<0.05 versus control plasmid only. ^#^p<0.05 versus IGFBP-3 NB plasmid. ^$^p<0.05 versus TNF-α. n=4, data are mean±SEM.

TNF-α can contribute to the induction of proinflammatory cytokines and adhesion molecules [35]. We examined potential cross-talk between IGFBP-3 and the TNF-α/ICAM-1 signaling cascade using the RECs in the high ambient glucose system. The RECs were grown in normal or high glucose and transfected with IGFBP-3NB or control plasmid DNA for 24 h. Some cells were also treated with TNF-α for 4 h after transfection. The results show IGFBP-3 overexpression inhibited TNF-α-induced ICAM-1 levels (Figure 3A). In Figure 3B, successful transfection with IGFBP-3 plasmid is shown.

### Intercellular adhesion molecule-1 small interfering ribonucleic acid inhibited tumor necrosis factor-alpha–induced adhesion in a flow-chamber assay

**Figure 4:**
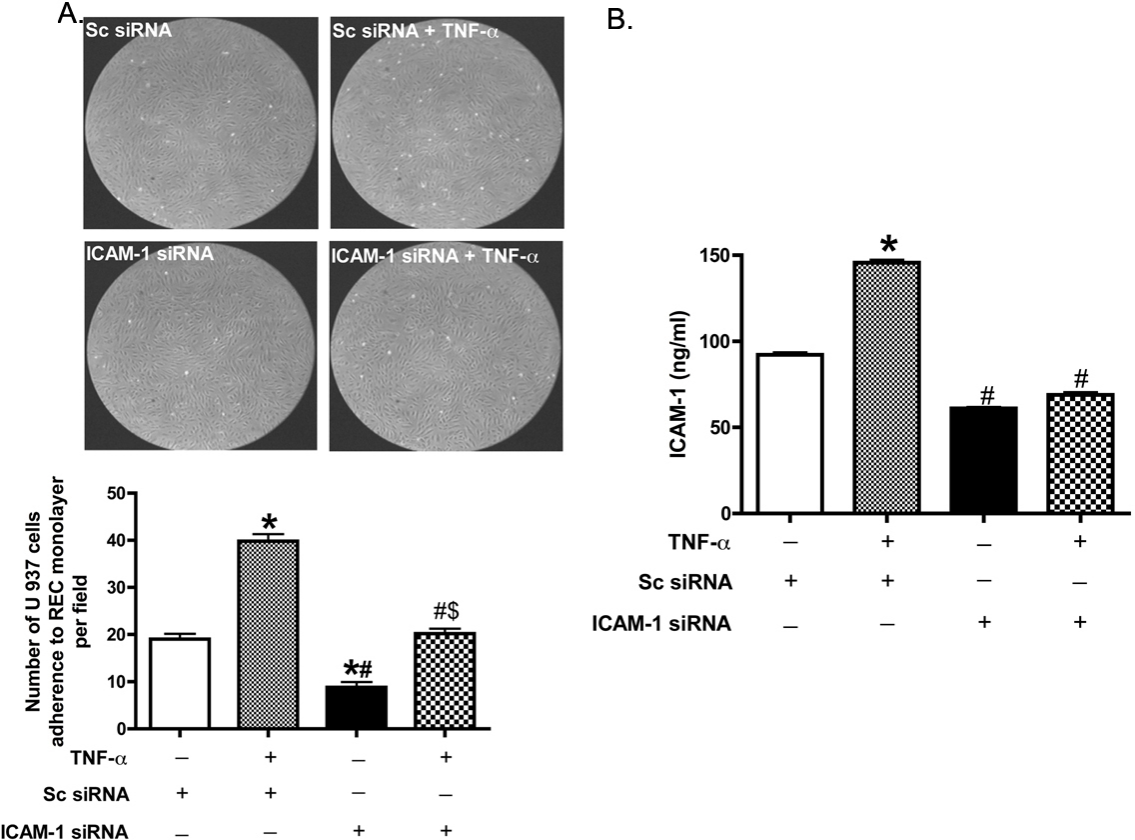
For both experiments, U937 cells were perfused over the retinal vascular endothelial cell (REC) monolayer culture in 25 mM glucose medium, which were transfected with scrambled siRNA or ICAM-1 siRNA for 24 h, followed by treatment with and without TNF-α for 4 h. Images were taken at the second hour after flow was initiated; data are expressed as the mean number of adhering cells/field ± SD for at least four different fields of view. **A**: ICAM-1 siRNA inhibited TNF-α induced monocytes adhesion to RECs in a flow chamber system. *p<0.05 versus Sc siRNA. ^#^p<0.05 versus Sc siRNA + TNF-α. ^$^p<0.05 versus ICAM-1siRNA. **B**: ELISA results of ICAM-1 concentration after ICAM-1 siRNA transfection following exposure to the flow chamber. *p<0.05 versus Sc siRNA. ^#^p<0.05 versus Sc siRNA + TNF-α. n=3, data are mean±SEM.

Since TNF-α can induce ICAM-1 expression and ICAM-1 promotes cellular adhesion [36,37], we wanted to measure whether the adhesion induced by TNF-α is mediated through ICAM-1. RECs grown in high glucose on the attachment factor-coated slides were transfected with ICAM-1 siRNA for 24 h and treated with TNF-α for 4 h. The slides were then mounted in a parallel-plate flow chamber and subjected to a continuous flow of U937 human monocytes (10^5^/ml) in culture medium, and images were taken at the second hour in the chamber. Figure 4 shows ICAM-1 siRNA inhibited TNF-α-induced monocyte adhesion to the REC monolayer in high ambient glucose.

### Insulin-like growth factor binding protein-3 inhibited tumor necrosis factor-alpha induced adhesion in a flow-chamber assay

**Figure 5:**
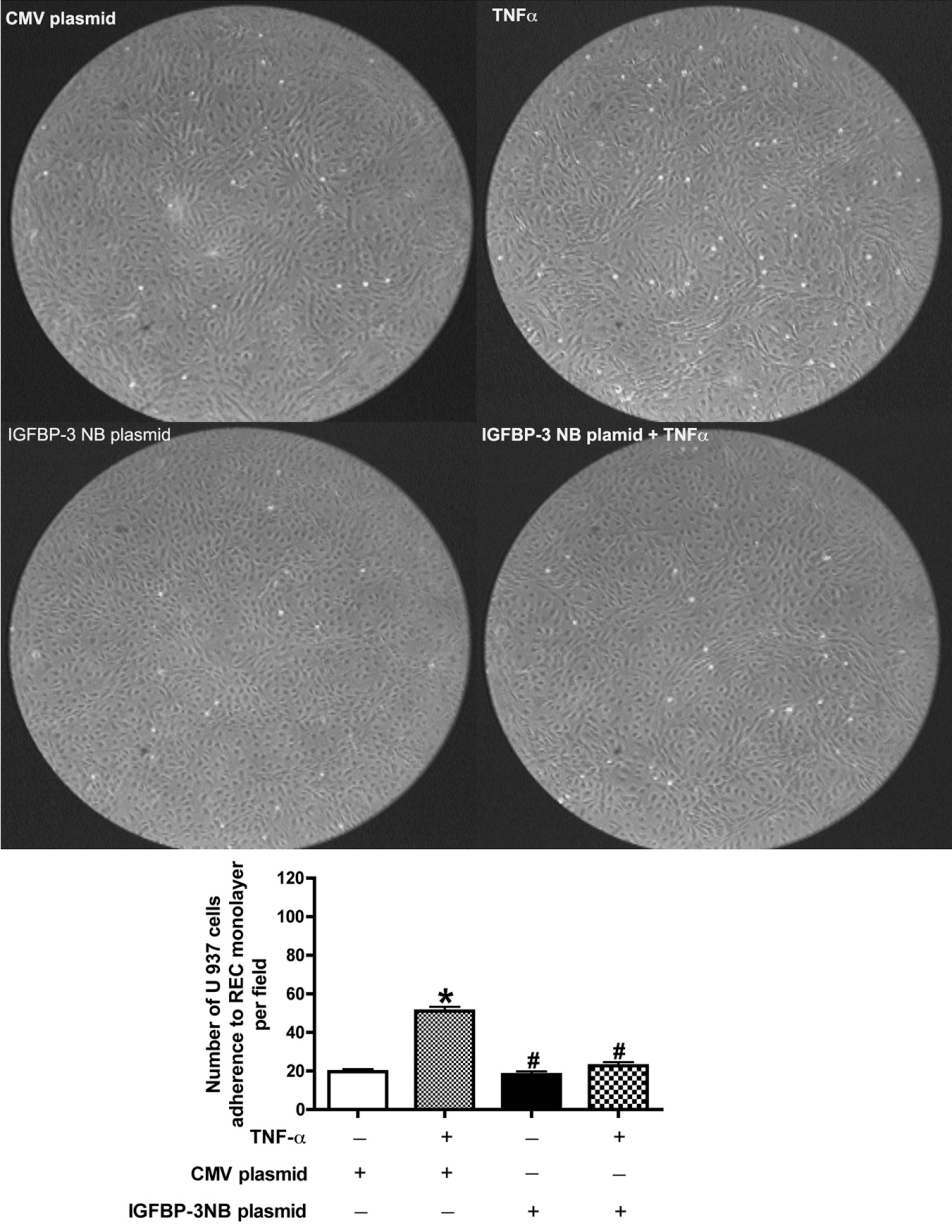
Insulin-like growth factor binding protein-3 (IGFBP-3) inhibited tumor necrosis alpha (TNF-α) induced monocytes adhesion to RECs in a flow chamber system. U937 cells were perfused over the REC monolayer, which were grown in 25 mM high glucose medium and transfected with control or IGFBP-3NB plasmid DNA for 24 h, following treatment with and without TNF-α for 4 h. Images were taken at the second hour after flow was initiated; data are expressed as the mean number of adhering cells/field ±SD for at least 4 different fields of view. *p<0.05 versus control plasmid. ^#^p<0.05 versus IGFBP-3 NB plasmid. ^$^p<0.05 versus control plasmid +TNF-α. n=3, data are mean±SEM.

In Figure 5, the effects of IGFBP-3 on TNF-α induced adhesion in high ambient glucose is compared. Cells were grown in high glucose medium and transfected with IGFBP-3NB or control plasmid DNA. For some groups, 24 h after transfection, cells were treated with TNF-α for 4 h. Results show that IGFBP-3 inhibited TNF-α-induced monocyte adhesion to the REC monolayer in high ambient glucose.

## Discussion

The inhibition of monocyte-REC adhesion by IGFBP-3 suggests that IGFBP-3 may have a role in regulating inflammation in diabetic retinopathy. We have previously demonstrated that IGFBP-3 inhibited REC apoptosis in cells cultured in high glucose and in samples from type 1 diabetic rat retinas [22]. These findings are supported by work on endometrial cancer cells demonstrating that IGFBP-3 is antimigratory [38]. Thus, we investigated IGFBP-3 actions on cell migration and adhesion invasive properties as they may relate to diabetic retinopathy.

The goal of this study was to characterize the effects of IGFBP-3 actions on REC-monocyte adhesion relevant in the progression of diabetic retinopathy, independent of any IGF-1 response. To address the goal of these studies, we used RECs transfected with IGFBP-3NB plasmid DNA in high ambient glucose to detect ICAM-1-mediated monocyte adhesion in the flow chamber. The results suggest that IGFBP-3 can decrease inflammatory response to TNF-α induced adhesion and subsequent expression of ICAM-1 on the endothelial cells. IGFBP-3 attenuation of monocyte adhesion suggests that restoring IGFBP-3 input can counterbalance the inflammatory response following high-glucose exposure.

IGFBP-3 is expressed, produced, and secreted by RECs leading to reduced apoptosis, adhesion, and vascular permeability. However, the detailed causal mechanisms remain unknown. The IGFBP-3 protein can bind to cell surface glycosaminoglycans or via specific molecules such as low-density lipoprotein-related protein in modulating apoptosis and tumor suppression [39]. The long-standing search for IGFBP-cell surface receptors has not yet provided definitive results, and a specific receptor for IGFBP-3 has not been confirmed in the retina. A report on the effects of IGFBP-3 on cell adhesion states that inhibition of endocytosis did not influence IGFBP-3 actions [40]. Researchers have shown that IGFBP-3 can inhibit the expression of nuclear factor-kappa B–regulated angiogenic factors and cell adhesion molecules such as ICAM-1 in tumorigenic prostate cell lines [41]. Other researchers reported that IGFBP-3 inhibited TNF-α-induced nuclear factor-kappa B activity in human colonic carcinoma cells [42]. However, the distinct mechanisms of how IGFBP-3 regulates cell adhesion need to be further analyzed.

In future studies, we will investigate the mechanisms by which IGFBP-3 regulates ICAM-1 and which steps of the migratory cascade are regulated by TNF-α and IGFBP-3. We also will further investigate the actions of IGFBP-3 when ICAM-1 is overexpressed in RECs.

In summary, these studies demonstrate that RECs stimulated by TNF-α activate ICAM-1 expression/presentation leading to increased cell adhesion. IGFBP-3 significantly inhibited cell adhesion and ICAM-1 levels in RECs. Our findings indicate that IGFBP-3 exerts antiadhesion effects via IGF-independent mechanisms that have cross-talk with TNF-α signaling. The use of IGFBP-3 as an anti-inflammatory agent in this manner may offer alternate means for treating diabetic retinopathy.
